# Supplementary material for: 2D/2D coupled MOF/Fe composite metamaterials enable robust ultra–broadband microwave absorption
Source: Nat Commun. 2024 Jul 5;15:5642. doi: 10.1038/s41467-024-49762-4 (PMC11226717; doi:10.1038/s41467-024-49762-4)
Supplement: Supplementary file 1 — Supplementary Information [file 41467_2024_49762_MOESM1_ESM.docx]

***Supporting Information for***

**2D/2D Coupled MOF/Fe Composite Metamaterials Enable Robust Ultra-Broadband Microwave Absorption**

Ning Qu^1, ‡^, Hanxu Sun^1, ‡^, Yuyao Sun^1^, Mukun He^1^, Ruizhe Xing^1^*, Junwei Gu^1^, and Jie Kong^1^*

^1^Shaanxi Key Laboratory of Macromolecular Science and Technology and MOE Key Laboratory of Materials Physics and Chemistry in Extraordinary Conditions, School of Chemistry and Chemical Engineering, Northwestern Polytechnical University, Xi’an 710072, P. R. China

**^‡^** These authors contributed equally: Ning Qu, Hanxu Sun.

*Corresponding Authors.

E-mail: kongjie@nwpu.edu.cn (J.K.), rzxing@nwpu.edu.cn (R.X.)

This PDF file includes:

Supplementary Note 1-7;

Supplementary Figures 1-16;

Supplementary Table 1-5;

Supplementary References;

**Supplementary Note 1. Materials:**

Cuprous oxide (Cu_2_O, 99.9%) and 4-Mercaptophenol (99%) were purchased from Shanghai TCI Ltd., China. The bisphenol-A epoxy resin (B-A EP) E-51, N, N-dimethylformamide (DMF, 99.9%) and ethanol (99.5 %) were supplied by from Shanghai Macklin Biochemical Technology Co., Ltd., China. The curing agent diethyltoluene diamine (DETDA, ≥98%) and the accelerator 2,4,6-tris((dimethylamino)methyl)phenol (DMP-30, ≥99.8%) were purchased from Shanghai Baika Chemical Co. Ltd., China. The defoamer organosilicone oil (OSO, 99%) and platinum-catalyzed liquid silicone rubber (LSR) were purchased from Longcheng Organic Silicone Chemical Co. Ltd., China. The spherical carbonyl iron (CI) particles were supplied by Shanghai Aladdin Reagent Co., Ltd., China. Multi-walled carbon nanotubes (MWCNTs) are provided by Kaisa New Materials Co., Ltd, Spherical copper powder (100 mesh) purchased from Beijing Innochem Technology Co., Ltd. All other chemical reagents (acetone (99.9%)) used in this study were provided by Sinopharm Group Chemical Reagents Co., LTD. All the reagents were used directly as received.

**Supplementary Note 2. Materials Characterizations:**

Field emission scanning electron microscope (FE-SEM, Zeiss SUPRA 55, Germany) and transmission electron microscope (TEM, JEM-2100F, 200 kV, Japan) were used to observe the morphology of all samples. The local microstructural details of the samples were further investigated using atomic force microscopy (AFM, MFP-3D-infinity, Asylum Research, USA). The structural information and elemental analysis of the samples were determined by X-ray diffraction (XRD, AXS D8, Bruker, Germany), Fourier transform infrared spectra (FT-IR, DSOR 27, Bruck, Germany), and X-ray photoelectron spectroscopy (XPS, PHI 500 VersaProbe^TM^, ULVAC-PHI, Japan). Magnetic hysteresis loop was measured by a vibrating sample magnetometer (VSM, VSM 7307, Lake Shore, USA). Thermogravimetric analysis (TGA, TGA 8000, PerkinElmer, USA) was performed in an argon atmosphere at a test range of 40 °C to 1000 °C to evaluate the thermal stability. The conductivity of the materials was obtained by analysis of a four-point probe surface resistance tester (MCP-T370, Loresta, Japan). Evaluation of nitrogen adsorption-desorption curves, porosity, and specific surface area by the Brunauer-Emmett-Teller method (BET, BeiShiDe3H-2000PS2, Beijing, China). The bulk density (ρ_0_) of the composite CuHT-FCIP-EP composites were measured by Archimedes’ method, while the apparent density of the metamaterial was calculated using the following equation: $\rho_{0}\times V_{r}$ (i.e., relative density, RD), where $V_{r}$ denotes the solid volume fraction of the metamaterial unit relative to the intact cell.

**Supplementary Note 3. Bandgap calculation:**

The Sc-MOF CuHT powder sample was tested by a solid-state UV-Vis diffuse reflectance spectrometer (PerkinElmer Lambda 950). The bandgap is calculated based on the Kubelka-Munk function^1^:

$$\begin{aligned} F\left( R \right)=\frac{K}{S}=\frac{\left( 1-R_{\infty} \right)^{2}}{2R_{\infty}}\#\left( 1 \right) \end{aligned}$$

$$\begin{aligned} E_{g}=\frac{1240\times m}{-b}\#\left( 2 \right) \end{aligned}$$

where $m$ and $b$ are obtained by a linear fit ($y=mx+$*b*) to the UV- Vis spectrum; in addition, it should be noted that $E_{g}$ has a unit of eV, $R$ refers to the reflectance, and $F(R)$ is proportional to the extinction coefficient ($\alpha$).

**Supplementary Note 4. Mechanical measurements:**

Compression measurements were carried out to evaluate the mechanical stability of the metamaterial absorber by means of an electronic universal testing machine (CMT5105, MTS, USA), and to ensure a quasi-static compression process, the loading speed of the testing machine was assigned to 0.5 mm/min. Three-point bending experiments of the composite materials were carried out by means of a SANS CMT4304 apparatus (Sans Materials Testing Co., Ltd., Shenzhen, China), in order to obtain the bending strength ($\sigma$) of the material, and the support distance between the two ends of the specimen as well as the loading speed were set to 30 mm and 0.5 mm·min^-1^, respectively, during the test.

**Supplementary Note 5. DFT calculations：**

We performed all density functional theory (DFT) calculations under the generalized gradient approximation (GGA) condition using the first principles tool, Vienna Ab initio Simulation Package (VASP)^2^, utilizing the Perdew-Burke-Ernzerhof (PBE)^3^ formulation. We have chosen the projected augmented wave (PAW) potentials^4, 5^ to characterize the ionic core and to take valence electrons into account using a plane-wave basis set with a kinetic energy cutoff of 450 eV. Using Gaussian smearing method and a width of 0.05 eV allows for partial occupancy of the Kohn-Sham orbitals. In order to optimize the geometry and lattice size, the Brillouin zone integration was performed with 4×3×1 Γ-centered k-point sampling^6^. The convergence energy threshold for the self-consistent calculations is 10^-5^ eV. The equilibrium geometries and lattice constants were optimized for a maximum stress of up to 0.02 eV·Å^-1^ per atom. A 15 Å vacuum layer is usually added to the surface to eliminate artificial interactions between periodic images. Weak interactions are described by the DFT+D3 method using empirical correction in the Grimme’s scheme^7, 8^. The spin polarization method was used to describe the magnetic system. In addition, input files and output data for charge difference and density of states (DOS) were generated by the Vaspkit^9^ tool. Considering the strong structural correlation effects of the transition metal, the structure optimization and electronic structure calculations were performed by using the spin-dependent GGA plus Hubbard correction $U$ method, with an effective $U_{eff}$ parameter of 3.4 eV for Fe atoms and 4.0 eV for Cu atoms.

**Supplementary Note 6. Electromagnetic simulation technology:**

CST STUDIO SUITE 2022 software is used to simulate the bistatic radar cross section (RCS) results of CuHT-FCIP-EP composites at specific frequencies. First, in the time-domain solver, according to the metal-back model, open boundary conditions are used in x, y, and z directions during the simulation, and then the perfect electric conductor (PEC) and absorber are modeled as squares, which are specifically represented as an ultrathin PEC layer (200 × 200 mm) and an absorber layer (200 × 200 × 2.25 mm). Linearly polarized plane EMWs incident from the positive direction of Z axis to the negative direction of Z axis, and the direction of electric polarization propagation is along the X-axis (the model plates are placed on the X-O-Y plane). Furthermore, the reflectivity, electric field distribution, magnetic field distribution and power loss density of the CuHT-FCI-EP composite metamaterial absorber are calculated by inputting the electromagnetic parameters of the composite material with dispersive properties into this simulation software package. The relevant simulation methods of the other structure-type absorbers are the same as mentioned above. In order to reduce the computational cost, the individual cells of the these structures are set as periodic boundaries, which are computed using a frequency domain solver and simultaneously endowed with a simulated electromagnetic source.

**Supplementary Note 7. Synthesis and characterization of CuHT:**

CuHT was obtained through the solvothermal reaction of 4-hydroxybenzenethiol with cuprous oxide. Scanning electron microscopy (SEM) images show that, unlike the previously reported CuHT crystals^10^, the CuHT synthesized in the present work has a graphene-like morphology. The average length (*L*) to width (*B*) ratio is about 1.70 (**Supplementary** **Fig. 1a**). Energy-dispersive spectroscopy (EDS) spectra reveal a uniform distribution of the Cu, C, S, and O elements (**Supplementary Fig. 1c**), which confirms the uniformity of the composition of the CuHT MOF crystal structure. The selected area electron diffraction (SAED) pattern shows that CuHT exhibits a typical polycrystalline state (**Supplementary Fig. 1d**). This is also evidenced by the corresponding X-ray diffraction (XRD) pattern (**Supplementary Fig. 1e**). X-ray photoelectron spectroscopy (XPS) results confirm the coexistence of the four elements (**Supplementary Fig. 1g**) and the corresponding valence states. In the C1*s* spectrum of CuHT, the two main peaks with binding energies of 286.2 and 284.7 eV correspond to the C–O and C–H bonds of the aromatic ring, respectively (**Supplementary Fig. 1h**), while the weak peak at 291.5 eV is attributed to the O=C–O, suggesting that the ligands may have been partially oxidized during synthesis. The S 2*p* fine spectrum shows a peak at 161.5 eV (S–Cu) (**Supplementary Fig. 1i**), which demonstrates the successful coordination of the S atoms to the Cu sites and the possibility of constructing an effective CuS network.


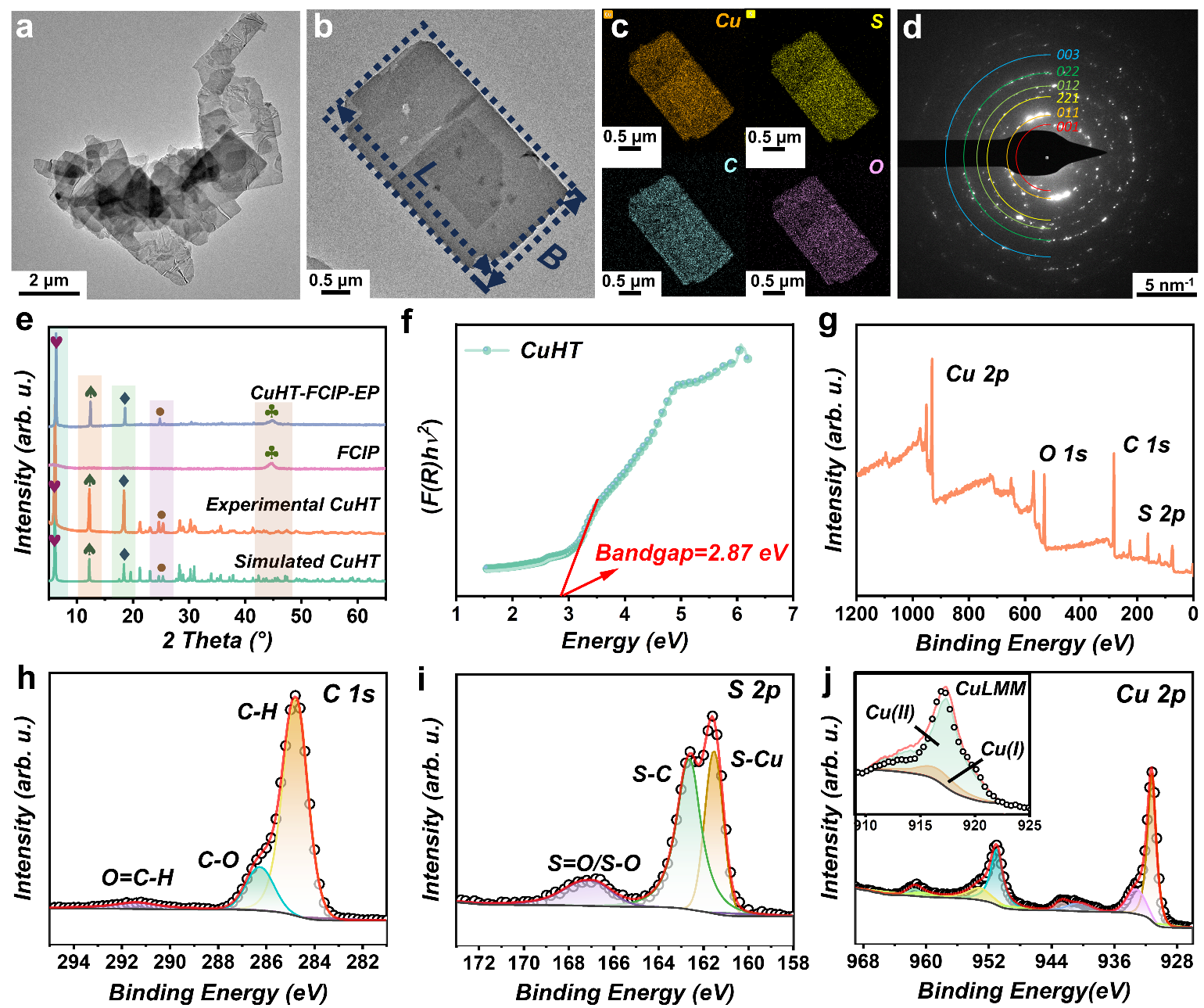
 **Supplementary Fig. 1 Characterization of CuHT.** **a-b** TEM images. **c** Elemental mapping of copper (Cu), carbon (C), sulfur (S), and oxygen (O) atoms. **d** The electron diffraction pattern of polycrystalline region. **e** X-ray diffraction (XRD) pattern. **f** The bandgap calculated based on the Kubelka-Munk function (**Equation. (1)**). **g-j** X-ray photoelectron spectroscopy (XPS) patterns.

In addition, **Supplementary Fig. 1j** shows two strong peaks with binding energies of 931.15 and 950.9 eV, which correspond to Cu 2*p*_3/2_ and Cu 2*p*_1/2_ in the sample, respectively, and the Cu LMM spectra (**Supplementary Fig. 1j, inset**) reveal the coexistence of Cu(II) and Cu(I) in CuHT. The band at about 3383 cm^−1^ observed in the Fourier-transform infrared spectroscopy (FT-IR) spectra corresponds to the O–H stretching vibration, which indicates that the hydroxyl groups have been retained during the synthesis of CuHT (**Supplementary Fig. 2a**). However, the O–H stretching vibration in phenol generally occurs at wave numbers higher than 3500 cm^−1^. It can be considered that the CuHT unit generates hydrogen bonds during the self-assembly process, causing the absorption peak to shift significantly. Therefore, CuHT is formed through the connection of three μ_3_-S atoms of the HT molecule with the Cu ions, their self-assembly into a honeycomb-shaped CuS layer, and their subsequent expansion in the corresponding plane. At the same time, the O–H∙∙∙O hydrogen bonding between the −OH groups of the HT molecule enable the layer-by-layer stacking of these CuS layers. Since the interactions between these weak hydrogen bonds can be considered electrostatic, we speculate that this may be the direct cause of the relatively uniform layered shape of CuHT.

We adopted the Brunner–Emmett–Teller (BET) theory to measure the specific surface areas (SSAs) and pore parameters of the CuHT powder samples. The N_2_ adsorption–desorption curves of the CuHT samples are typical IV-type curves (**Supplementary Fig. 2b**), and the SSA of CuHT is 14.113 m^2^·g^−1^ with a pore size of ~8 nm, which confirms that CuHT has a mesoporous structure (**Supplementary Fig. 2c**). The presence of a mesoporous structure is believed to enhance the impedance matching and contribute to EMW loss^11^. The thermogravimetry (TG) results reveal that the CuHT skeleton has a good thermal stability and can remain stable at 232 °C (**Supplementary Fig. 2d**), which ensures the stability of CuHT as an EMW-absorbing material in practical applications.

**
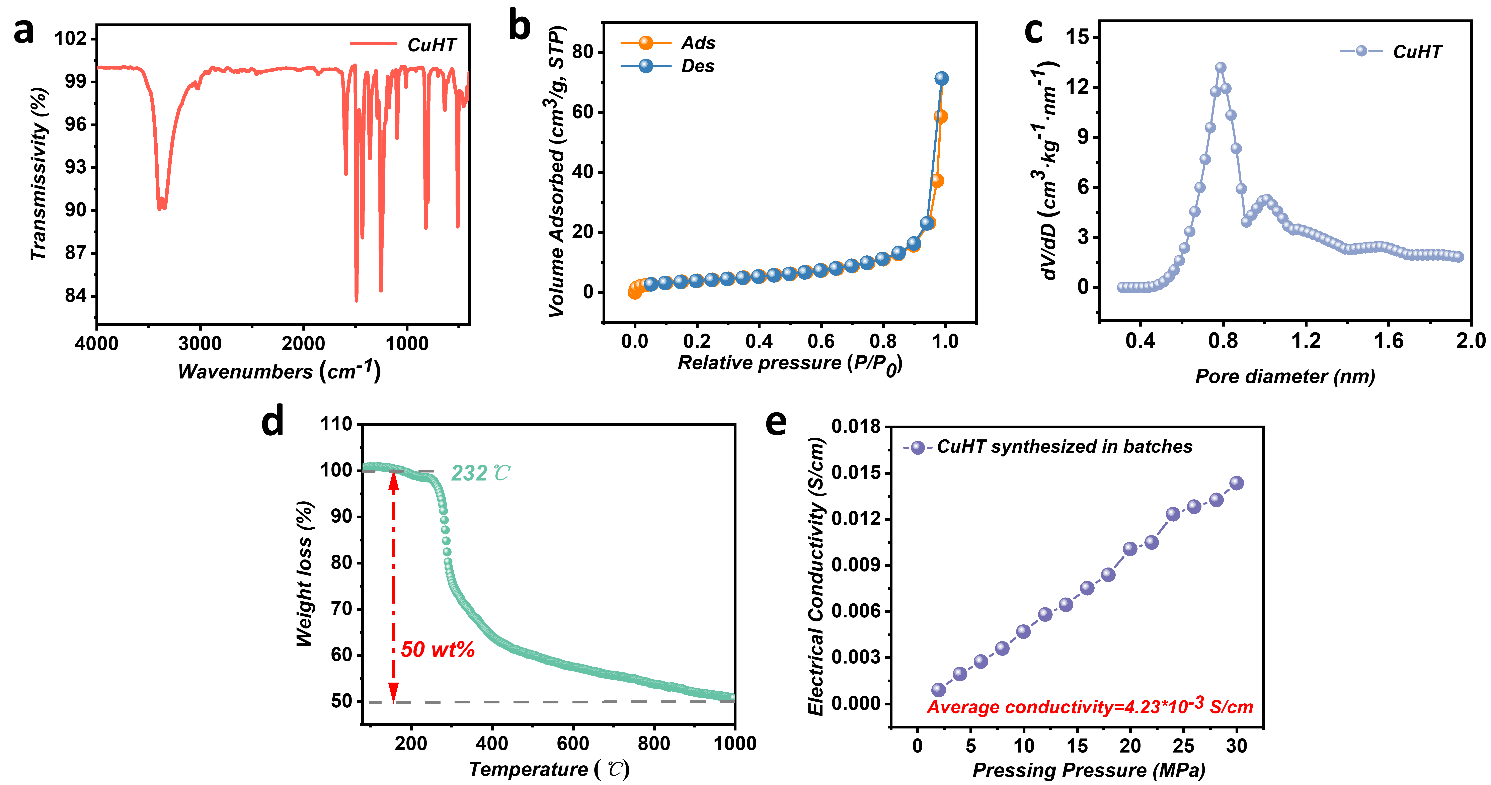
**

**Supplementary Fig. 2** **Other physical or chemical properties of CuHT.** **a** Fourier transform infrared spectroscopy (FTIR) of CuHT powder. **b** N_2_ adsorption-desorption isotherms and **c** pore size distribution. **d** The thermogravimetric analysis (TGA). **e** Conductivity curve.

In addition, the electrical conductivity is a key parameter that affects the performance of EMW-absorbing materials as it significantly influences the resistive loss capability of the material as well as its polarization relaxation. In this work, CuHT is characterized by an infinite layered Cu–S 2D network in its interior, which combined with the high interlayer contact area provided by the 2D material, endows CuHT with attractive electrical conductivity properties. To confirm this, we measured the resistivity of the CuHT powder using the four-probe method and found that its conductivity could reach 4.23×10^−3^ S·cm^−1^ (**Supplementary Fig. 2e**), which falls within the threshold range of EMW-absorbing materials (10^−6^–10^0^ S·cm^−1^)^12^. This unique charge transport mechanism may be related to the graphene-like honeycomb structure inside CuHT. Solid-state UV–Vis diffuse reflection results show that CuHT has a large optical band gap of 2.87 eV (**Supplementary Fig. 1f**) and thus exhibits typical semiconducting properties. In addition, CuHT has an ultra-low density, with a natural packing density of only 0.0856 g·cm^−3^, which is 64.18% of that of carbon nanotubes and only 2.96% of that of metallic Cu powder. The unique semiconducting properties and 2D layered structure of CuHT, in combination with its low weight, make this class of materials highly promising EMW-absorbing materials.

**
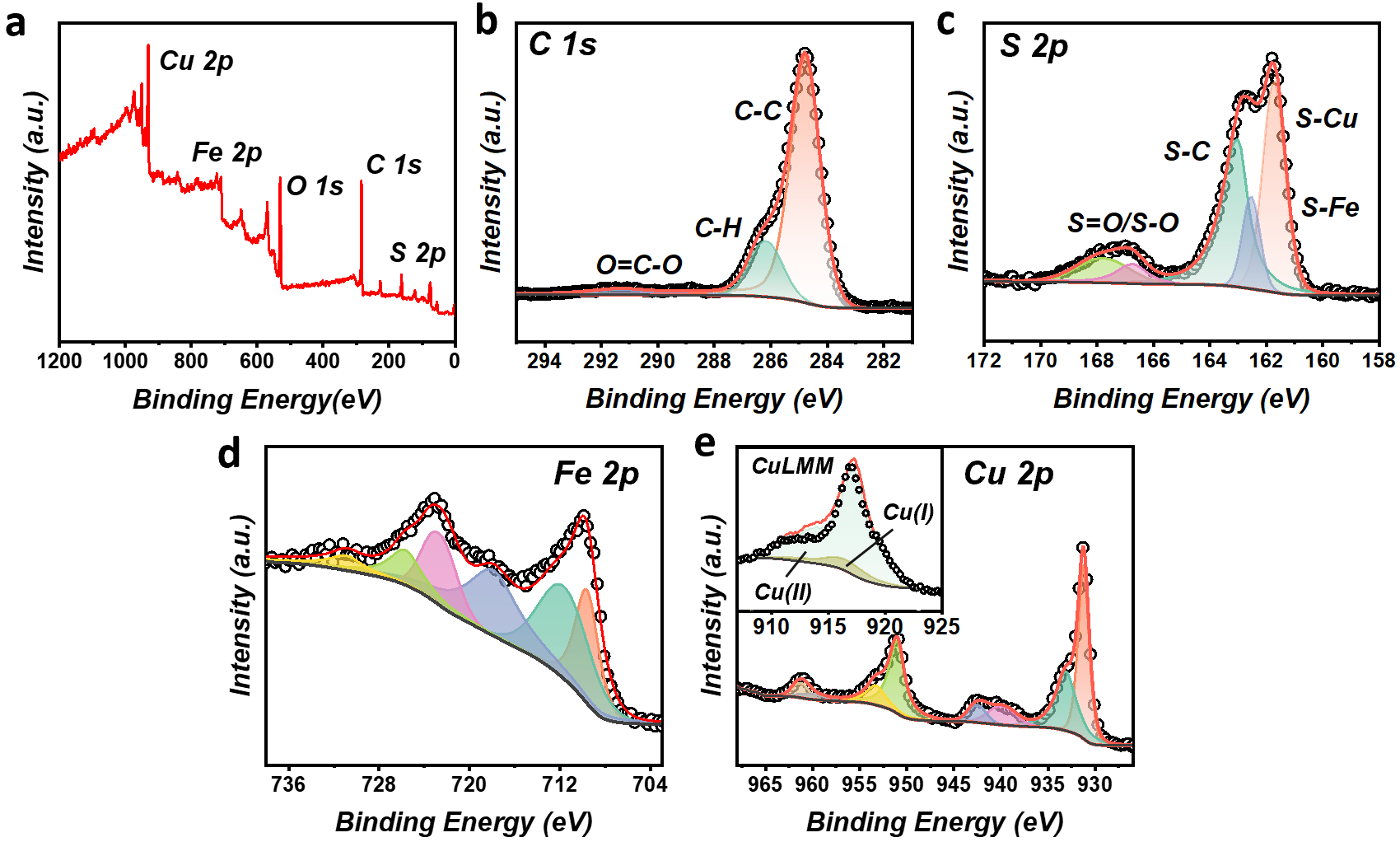
**

**Supplementary Fig. 3 The XPS survey spectra of CuHT-FCIP composites. a** elemental survey map. high-resolution XPS spectra of C 1s **b**, S 2p **c**, Fe 2p **d**, Cu 2p **e** and Cu LMM **e inset** of CuHT-FCIP composites.

In order to investigate the internal elements and the corresponding chemical states of the CuHT-FCIP composites, they were characterized by XPS. As shown in the XPS measurement spectra (**Supplementary Fig. 3a**), the elements Cu, Fe, C, S, and O coexist in the sample. In addition, partial oxidation of the ligand of CuHT resulted in a peak with a binding energy of 291.3 eV (**Supplementary Fig. 3b**). Most importantly, the S *2p* fine spectra of CuHT shows the presence of S-Fe at a binding energy of 126.6 eV (**Supplementary Fig. 3c**), representing the strong interaction between CuHT and FCIP during sonication. **Supplementary Fig. 3d** demonstrates that the high-resolution Fe *2p* spectra of the composites are divided into six main peaks, with two peaks at 709.8 eV and 712.3 eV attributed to Fe 2p_3/2_, and the peaks fitted at 723.06 eV and 725.9 eV indexed as Fe 2p_1/2_. The two peaks at 718.3 and 731.48 eV are usually characteristic of satellite peaks of Fe. **Supplementary Fig. 3e** demonstrates that the interaction or interface effect between FCIP and CuHT has no significant influence on the electron energy level of Cu element in MOF structure.


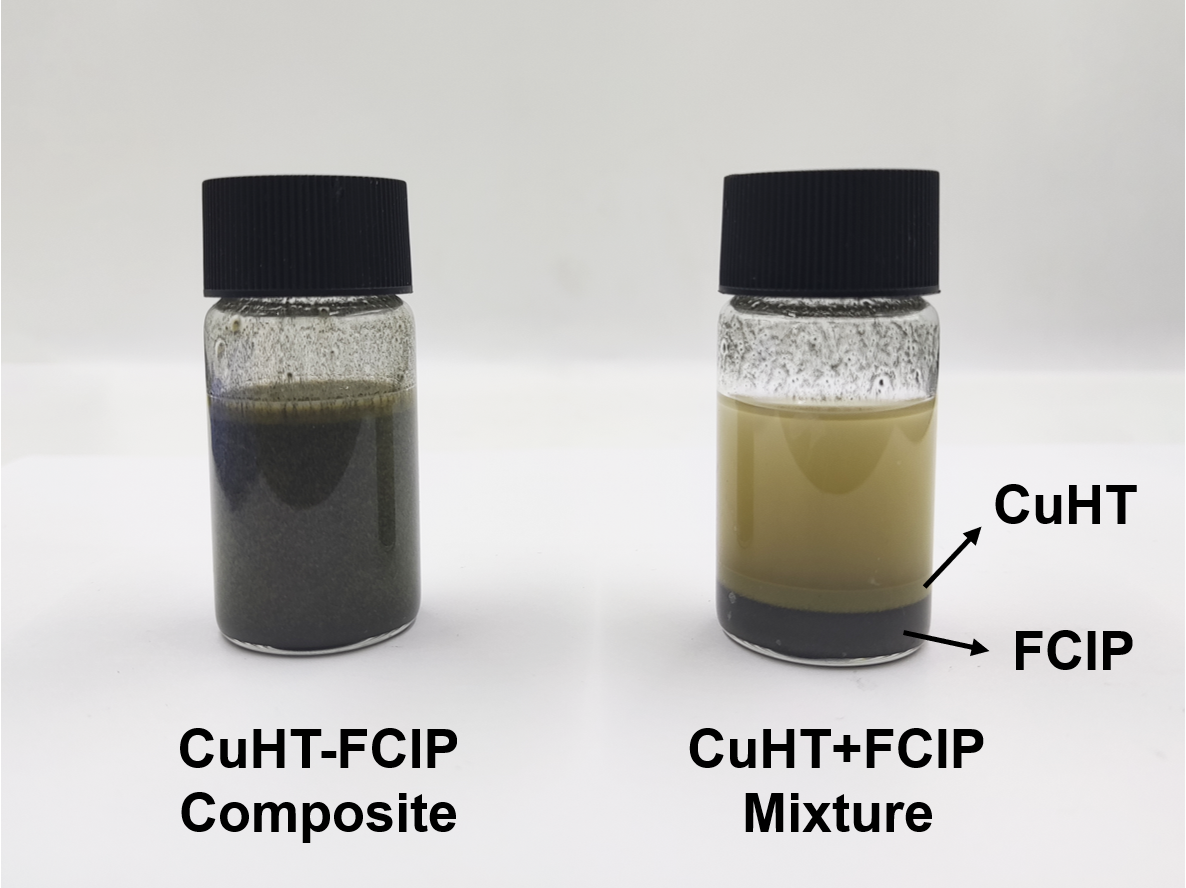


**Supplementary Fig. 4** Photos of as-prepared CuHT-FCIP composite by sonication (left) and the dispersion of CuHT and FCIP mixture by vortex mixing (right) after settling for 1 min.

Compared to simply mixing CuHT and FCIP, the CuHT-FCIP composite exhibits better dispersibility in ethanol solution after sonication. The main reasons are: first, the stronger power provided by ultrasonication causes the separation of the agglomerated FCIP and CuHT layers, thereby enhancing their inherent dispersibility; second, the ultrasonic power causes the dissolution of Cu ions on the surface of CuHT, resulting in the generation of uncoordinated thiol groups (-SH), which readily bond with partially oxidized Fe(II) or Fe(III) on the surface of FCIP, thus forming a sandwich-like CuHT-FCIP-CuHT assembly. Therefore, significant stratification is not observed in the CuHT-FCIP solution.

**
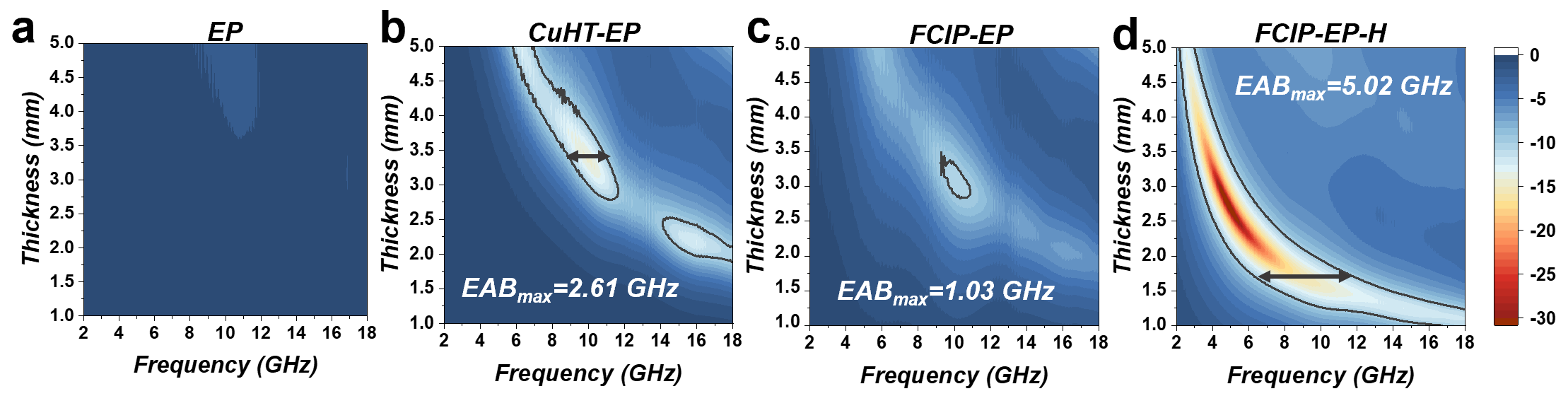
**

**Supplementary Fig. 5 EMW absorption performance of different samples.** 2D contour of RL values versus frequency at different thicknesses of **a** EP, **b** CuHT-EP, **c** FCIP-EP, **d** FCIP-EP-H.

**Supplementary Table 1** Composites consisting of different ratios of Sc-MOF CuHT, FCIP and EP.

| **Number** | **Weight Ratio** |
| --- | --- |
| EP | CuHT:FCIP:EP=0:0:10 |
| CuHT-FCIP-EP-S1 | CuHT:FCIP:EP=4:2:4 |
| CuHT-FCIP-EP-S2 | CuHT:FCIP:EP=3:3:4 |
| CuHT-FCIP-EP-S3 | CuHT:FCIP:EP=3:4:3 |
| CuHT-EP | CuHT:FCIP:EP=3:0:7 |
| FCIP-EP | CuHT:FCIP:EP=0:3:7 |
| FCIP-EP-H | CuHT:FCIP:EP=0:7:3 |


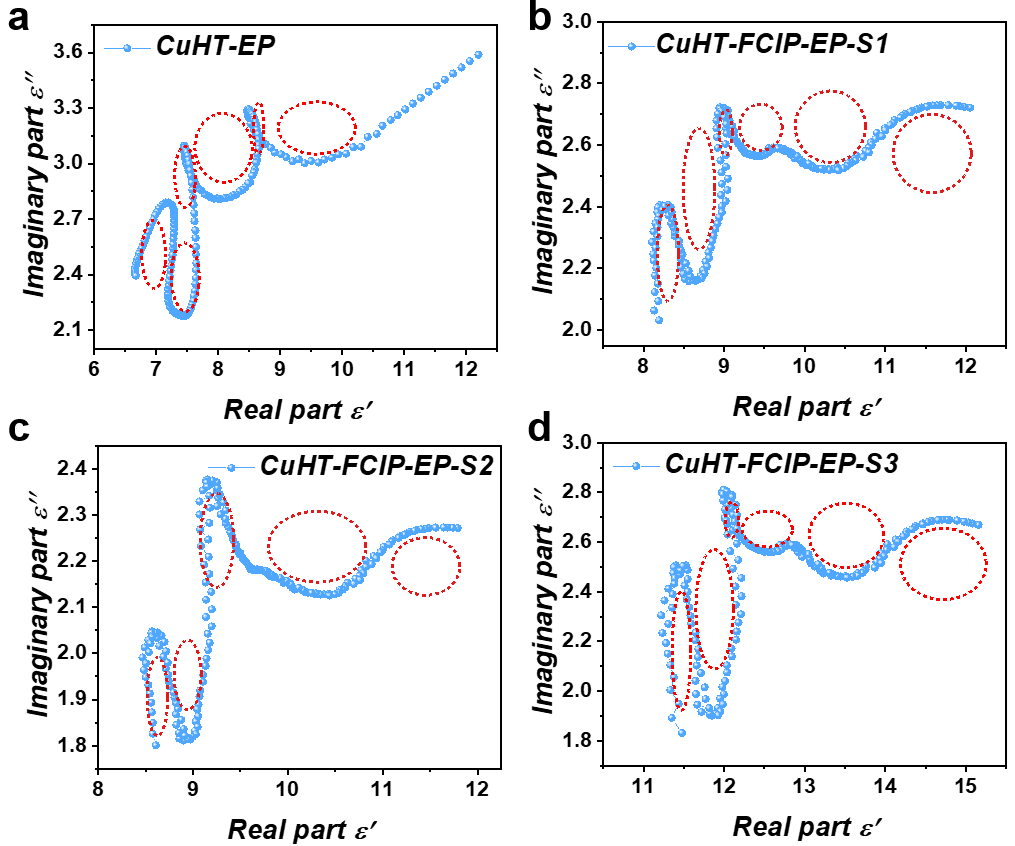


**Supplementary Fig. 6** Cole–Cole plots of **a** CuHT-EP, **b** CuHT-FCIP-EP-S1, **c** CuHT-FCIP-EP-S2 and **d** CuHT-FCIP-EP-S3. A curved line (indicated by red dashed circles) means polarization happens at the corresponding frequency and a straight line refers to the conduction behavior.

In the high-frequency region, the CuHT-EP samples with only dielectric assembly exhibit a linear relationship between ε' and ε'' suggesting a significant conductive loss process. However, in the remaining three CuHT-FCIP-EP composites where magnetic assembly is introduced, multiple relaxation processes occur under an alternating electromagnetic field, leading to a relative weakening of conductivity loss within the material. This indicates a notable enhancement of interfacial polarization and dipole polarization attributed to defects, oxygen-containing functional groups, and heterogeneous structures.


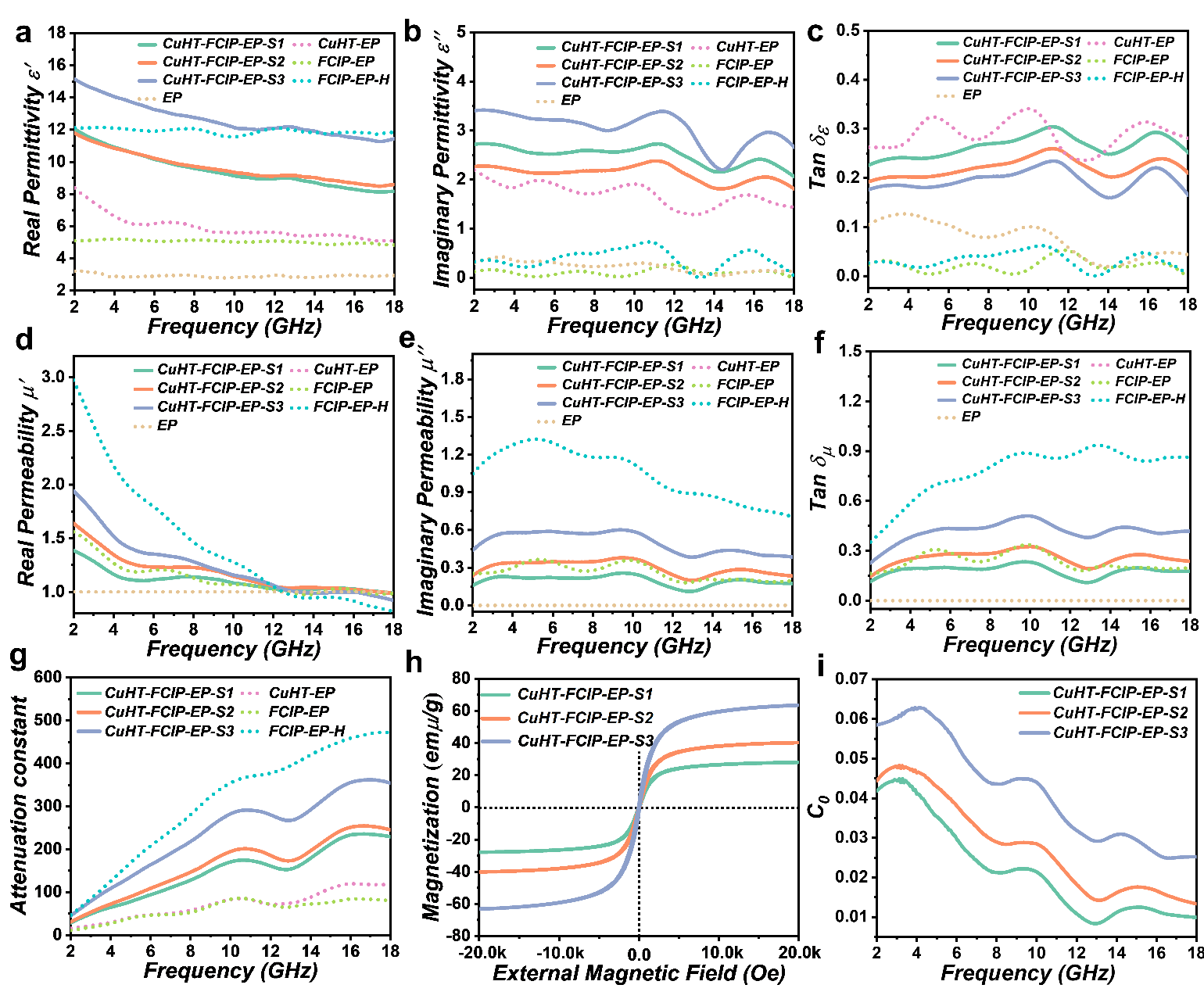


**Supplementary Fig. 7 Electromagnetic properties of CuHT-FCIP-EP composite.** Frequency dependence of **a** Real permittivity, **b** Imaginary permittivity, **c** $\tan\delta_{ɛ}$ **d** Real permeability, **e** Imaginary permeability and **f** $\tan\delta_{\mu}$ of S1, S2, S3, CuHT-EP, FCIP-EP, FCIP-EP-H and EP. **g** Attenuation constant of S1, S2, S3, CuHT-EP, FCIP-EP and FCIP-EP-H. **h** Magnetic hysteresis loops of S1, S2, S3 at room temperature. **i** Frequency dependence of $C_{0}$ for S1, S2, S3.

First, as shown in **Supplementary Fig. 7a, b**, benefiting from the rational design of the sandwich-type composites, the real and imaginary parts of the dielectric constants of S1-S3 reach a nearly ideal equilibrium. This is something that the rest of the control components could not have, although the CuHT-FCIP-H with high filling amount of FCIP. The trend of $\tan\delta_{ɛ}$ value is positively correlated with the concentration of the dielectric components, indicating an increase in the dielectric loss (**Supplementary Fig. 7c**). However, the mismatch between the dielectric response and the ultimate EMW loss capability then means that the role of magnetic losses cannot be ignored. It is noteworthy that S3 has a relatively high magnetic response but poor wave absorption performance (**Supplementary Fig. 7d-f**), which simultaneously proves that unilateral enhancement of the magnetic property contribution is ineffective. This is further evidenced by FCIP-EP-H, which has the highest real and imaginary parts of the magnetic permeability, especially the attenuation constant (**Supplementary Fig. 7g**), but similarly has far less EMW absorption performance than S2. Thus, our success in constructing the magnetoelectric balance mechanism within the composites again proved to be particularly necessary and critical.

The composites with sandwich-type structures all exhibit a pronounced ferromagnetic response (**Supplementary Fig. 7h**). The resolved saturation magnetization ($M_{s}$) values of the samples are 27.9, 40.3, and 63.7 emu/g for the CuHT/FCIP relative mass ratios of 4:2, 3:3, and 3:4, respectively. The corresponding coercivities ($H_{c}$) are almost the same, which implies that these materials are able to maintain a more stable magnetization state in the presence of an applied magnetic field. The $C_{0}$ curve ($C_{0}= \mu^{''}\left( \mu^{'} \right)^{-2}\left( f \right)^{-1}$)^13^ further reveals that the magnetic loss effect mainly originates from the natural resonance and exchange resonance (**Supplementary Fig. 7i**).


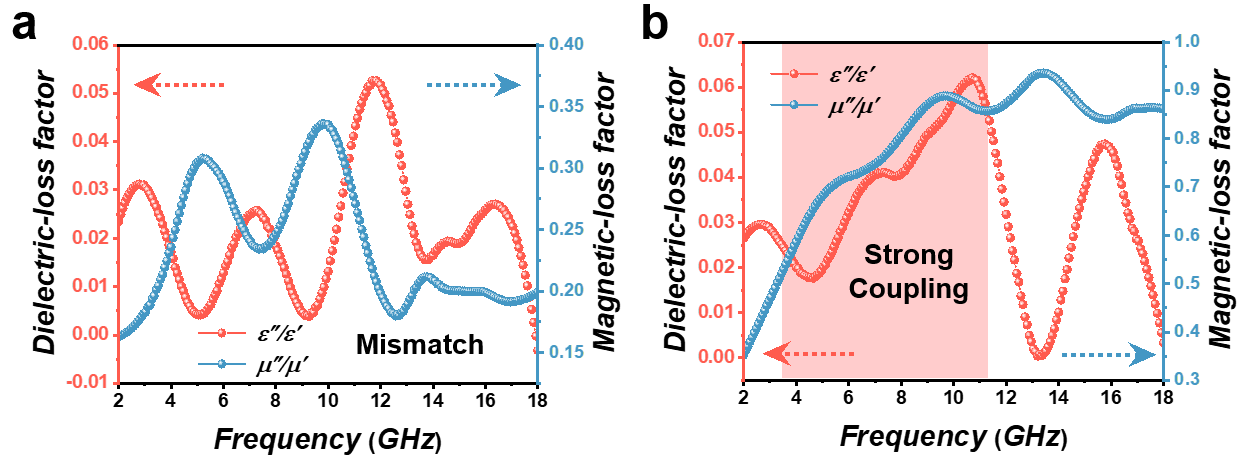


**Supplementary Fig. 8** Dielectric and magnetic loss curves of **a** FCIP-EP, **b** FCIP-EP-H.

The coupling state of pure FCIP resin is illustrated in **Supplementary Fig. 8**. The electromagnetic loss curve of FCIP-EP (with a FCIP to EP mass ratio of 3:7) exhibits a noticeable mismatch. This mismatch arises from the FCIP's low filling level, which prevents the formation of an effective conducting path within the material. Consequently, the dielectric loss is limited and unable to synergize adequately with the relatively strong magnetic loss. This shortfall is addressed by increasing the FCIP filling (FCIP-EP-H, with a FCIP to EP mass ratio of 7:3). However, despite this adjustment, the magnetic effect continues to dominate due to the absence of further reinforcement from the dielectric assemblies. As a result, the FCIP-EP-H only achieves partial coupling in the mid-to-low frequency region (<11 GHz).


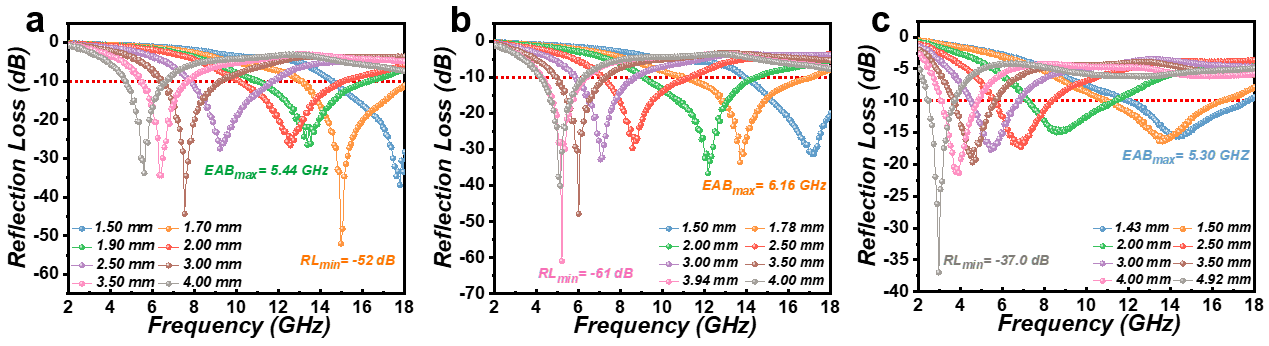


**Supplementary Fig. 9** Reflection loss of 2D CuHT-FCIP-EP at different thicknesses for **a** CuHT-FCIP-EP-S1, **b** CuHT-FCIP-EP-S2 and **c** CuHT-FCIP-EP-S3.

The CuHT/FCIP ratio significantly impacts both the position of the minimum absorption peak and the effective absorption bandwidth (EAB) of the composite materials. For dielectric-type electromagnetic absorption materials, the thickness of the absorber and its strongest absorption frequency satisfy the following equation:

$$\begin{aligned} d=\frac{c}{4f\sqrt{\varepsilon_{r}\mu_{r}}} \#\#(3) \end{aligned}$$

where $d$ is the thickness of the absorber, $\varepsilon_{r}$ is the complex permittivity, $\mu_{r}$ is the complex permeability, $f$ is the frequency, and $c$ is the speed of electromagnetic waves in vacuum. At lower thicknesses, sample S1-S3 exhibit significant absorption only in the high-frequency range (Ku band). As the thickness increases, the EAB gradually shifts towards lower frequency ranges. Meanwhile, at the same thickness, the strongest absorption peak frequencies of S1-S3 decrease sequentially. For example, when the thickness is 4 mm, the minimum absorption peak position for S1 is 5.82 GHz, for S2 it is 5.03 GHz, and for S3 it is 3.96 GHz. The optimal EAB of S1-S3 is 5.44 GHz, 6.16 GHz, and 5.30 GHz at thicknesses of 1.90 mm, 1.78 mm, and 1.43 mm, respectively. This is mainly because, firstly, compared to S1, both S2 and S3 contain a larger amount of magnetic components (FCIP), resulting in a higher overall refractive index ($n=\sqrt{\varepsilon\mu}$); secondly, sample S2 exhibits a magneto-dielectric coupling loss exactly in the 6-18 GHz range, thus enhancing its absorption capability within this range. Hence, the ratio of CuHT to FCIP significantly influences the absorption performance of the composite materials, providing a material foundation for the design of multilayer metamaterials.

**Supplementary Table 2** Comparison of the absorption performance of different MOF-derived composite materials.

| **Sample** | **Rl_min_ (dB)** | **EAB (GHz)** | **Thickness (mm)** | **Ref.** |
| --- | --- | --- | --- | --- |
| NPC/Co/CoO | −48.97 | 5.36 | 2.00 | 14 |
| HCSC/CNTs-30 | −41.60 | 4.90 | 2.00 | 15 |
| Co@NCMs | −50.40 | 3.85 | 2.70 | 16 |
| Cu/NC@Co/NC-3.75 | −54.13 | 5.19 | 2.50 | 17 |
| CoFe alloys@ZnO@C | −40.63 | 5.84 | 2.20 | 18 |
| CZC@M | −41.75 | 4.72 | 2.40 | 19 |
| Carbon/Co/Co_3_O_4_/CNTs/RGO | −59.20 | 5.70 | 2.00 | 20 |
| CoMn@CN | −39.90 | 5.24 | 2.00 | 21 |
| CoFe alloy@C | −40.00 | 5.62 | 2.00 | 22 |
| CuHT-FCIP-EP-S1 | −52.00 | 5.44 | 1.90 | This work |
| **CuHT-FCIP-EP-S2** | −**61.00** | **6.16** | **1.78** | **This work** |
| CuHT-FCIP-EP-S3 | −37.00 | 5.68 | 1.43 | This work |

**
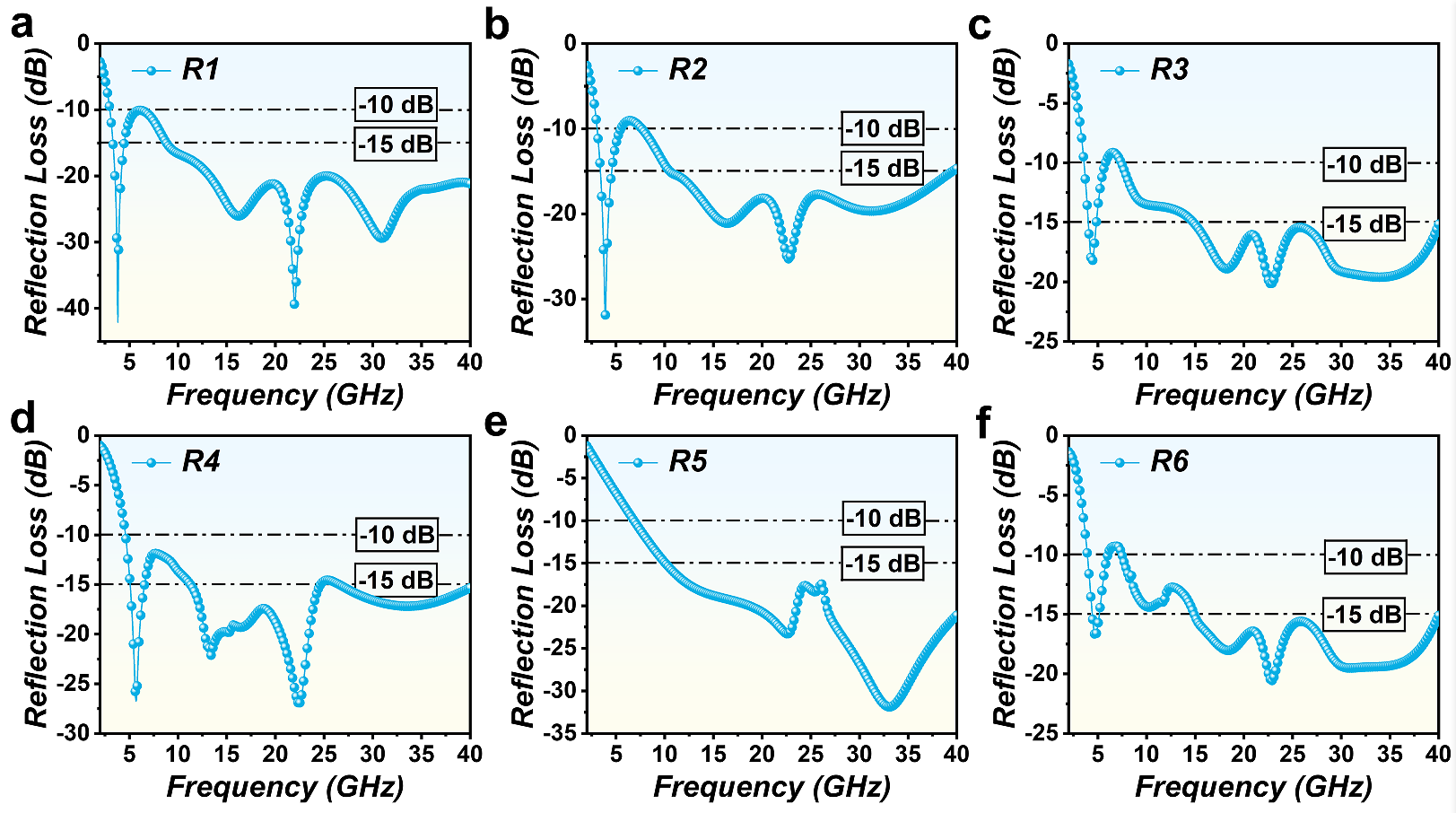
**

**Supplementary Fig. 10 Effect of different material combinations on the bandwidth of metamaterials.** The simulated reflectivity of metamaterial absorbers composed of different sequences R1 (a), R2 (b), R3 (c), R4 (d), R5 (e), R6 (f) in the frequency range of 2-40 GHz. (Detailed stacking sequence were listed in Supplementary Table 3)

**Supplementary Table 3.** CuHT-FCIP-EP metamaterials based on different material combinations.

| **ID** | **R1** | **R2** | **R3** | **R4** | **R5** | **R6** |
| --- | --- | --- | --- | --- | --- | --- |
| **Top layer** | S1 | S2 | S1 | S3 | S3 | S2 |
| **Middle layer** | S2 | S1 | S3 | S1 | S2 | S3 |
| **Bottom layer** | S3 | S3 | S2 | S2 | S1 | S1 |

All possible three-layer combinations are detailed in **Supplementary Table 3**. Comparing the simulated reflectivity results between R1, R2, and other combinations, it is evident that when S3 is placed at the bottom layer, the metamaterial exhibits superior absorption performance in the low-frequency range (2-5 GHz). This can be attributed to the higher content of strong magnetic component in S3, which maximizes the absorption of low-frequency EMWs. Further comparison between R1 and R2 results shows that, despite similar -10 dB EAB, when S1 is the top layer, the resonance absorption peak intensities of the metamaterial are significantly higher compared to the scenario when S2 is at the top. This is because S1 material has relatively lower attenuation constant, resulting in a gradient distribution from top to bottom. Such a distribution has been widely acknowledged to enhance the efficiency of electromagnetic absorption. Consequently, the combination of S1-S2-S3 was chosen as the optimal configuration.

**
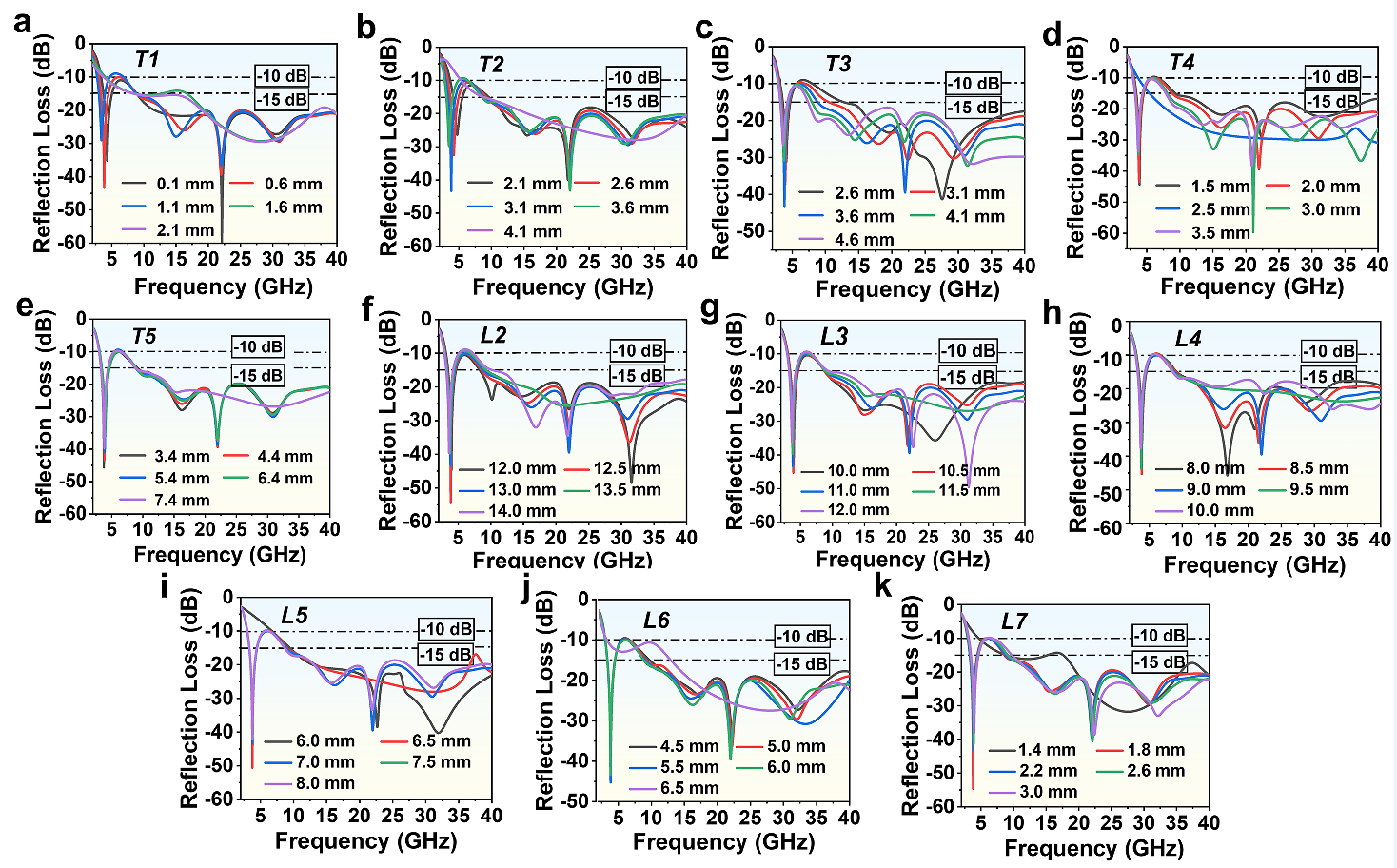
**

**Supplementary Fig. 11 Influence of structural dimensions on reflection loss of metamaterial absorbers.** By changing **a** T1, **b** T1, **c** T3, **d** T4, **e** T5 , **f** L2 , **g** L3 , **h** L4 , **i** L5 , **j** L6 and **k** L7 optimize the reflectivity in the frequency range from 2 to 40 GHz to determine the unit size of the absorber.

**Supplementary Table 4** Optimized dimension for the proposed metamaterial absorber unit.

| **Parameter** | **T1** | **T2** | **T3** | **T4** | **T5** | **L1** |
| --- | --- | --- | --- | --- | --- | --- |
| **Value** | 0.6 mm | 3.1 mm | 3.6 mm | 2.0 mm | 4.4 mm | 15.0 mm |
| **Parameter** | **L2** | **L3** | **L4** | **L5** | **L6** | **L7** |
| **Value** | 13.0 mm | 11.0 mm | 9.0 mm | 7.0 mm | 6.0 mm | 2.2 mm |

Based on the permittivity and permeability of CuHT-FCIP-EP-S1, CuHT-FCIP-EP-S2, and CuHT-FCIP-EP-S3 (**Supplementary Fig. 6**), we calculated the reflection loss curves of the multilayered composite metamaterials with different geometry parameters by CST STUDIO SUITE 2022 ^23^. Floquet boundary conditions were used in this process, the frequency range was set to 2-40 GHz, and the structure was meshed in a hexahedral form. As shown above, the variation of the layer thicknesses T1-T4 has a significant effect on the position of the absorption peaks and the absorption depth of the absorber, with the increase in thickness enhancing the resonant electrical length of the absorber (**Supplementary Fig. 11a-d**) and the position of the absorption peaks gradually shifted towards the lower frequency range. Unfortunately, this also consequently caused a serious sacrifice of reflectivity in the 4-8 GHz frequency range, but T5 had less effect on the trend of reflectivity (**Supplementary Fig. 11e**). In addition, when L2-L6 are increased in steps of 0.5 mm, respectively (**Supplementary Fig. 11f-j**), the absorption peak position and appearance are seriously affected. However, for L7, the absorption peak depth of the absorber in the low-frequency band gradually decreases when increasing in steps of 0.4 mm (**Supplementary Fig. 11k**), especially when the size of L7 is less than 1.4 mm, the trend of the reflection loss in the ranges of 2-4 GHz and 20-40 GHz varies drastically, which suggests that the edge length of the top honeycomb-like structural plays an important role in the impedance matching of the absorber. Finally, a set of parameters were determined as summarized in **Supplementary Table 4**.

**
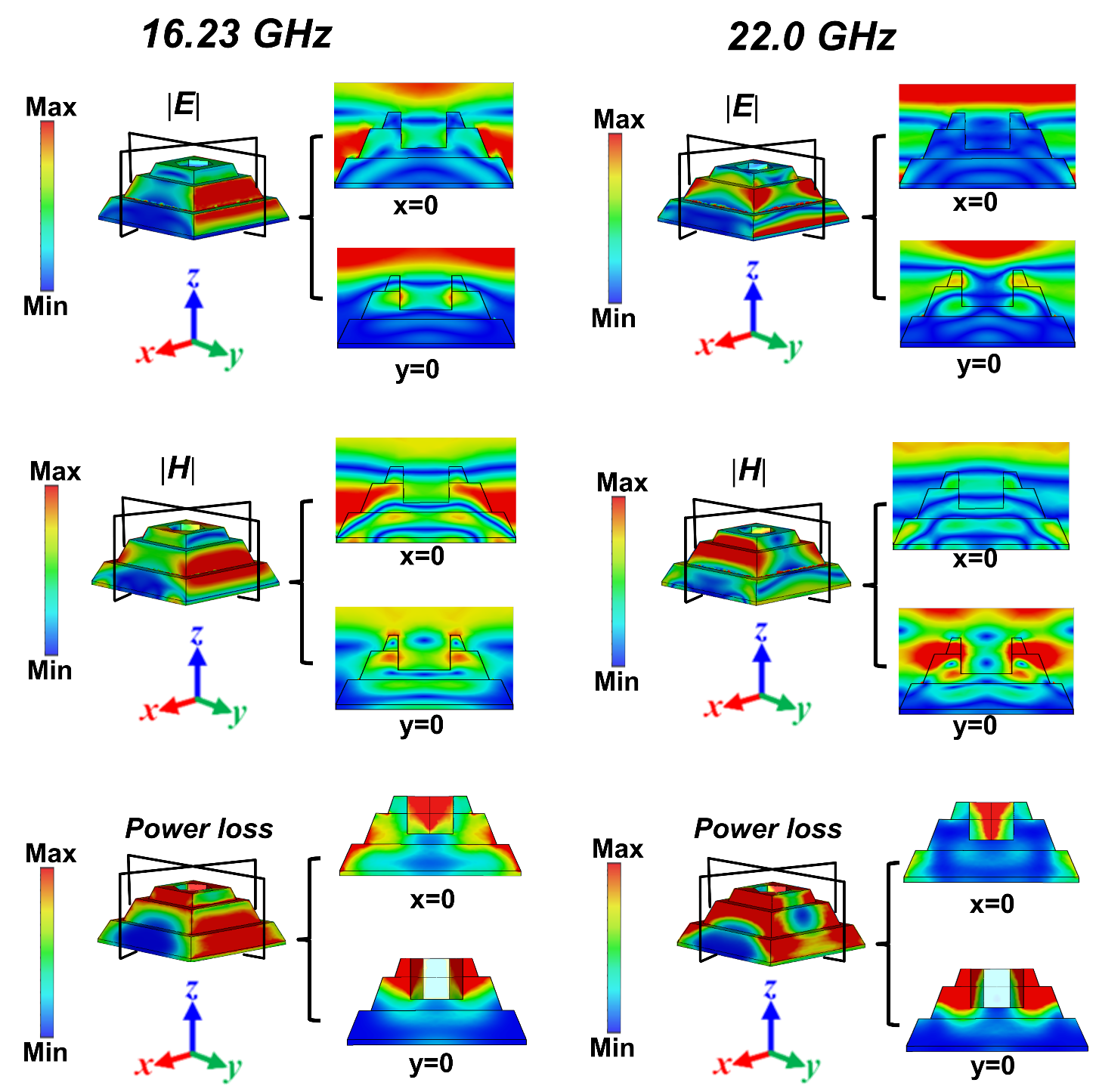
 Supplementary Fig. 12 Electromagnetic simulation of metamaterial absorbers.** Distributions of the electric field, magnetic field, and power loss density of the metamaterial absorber at different absorption peak frequencies: 3.82 GHz, 22.0 GHz.

**
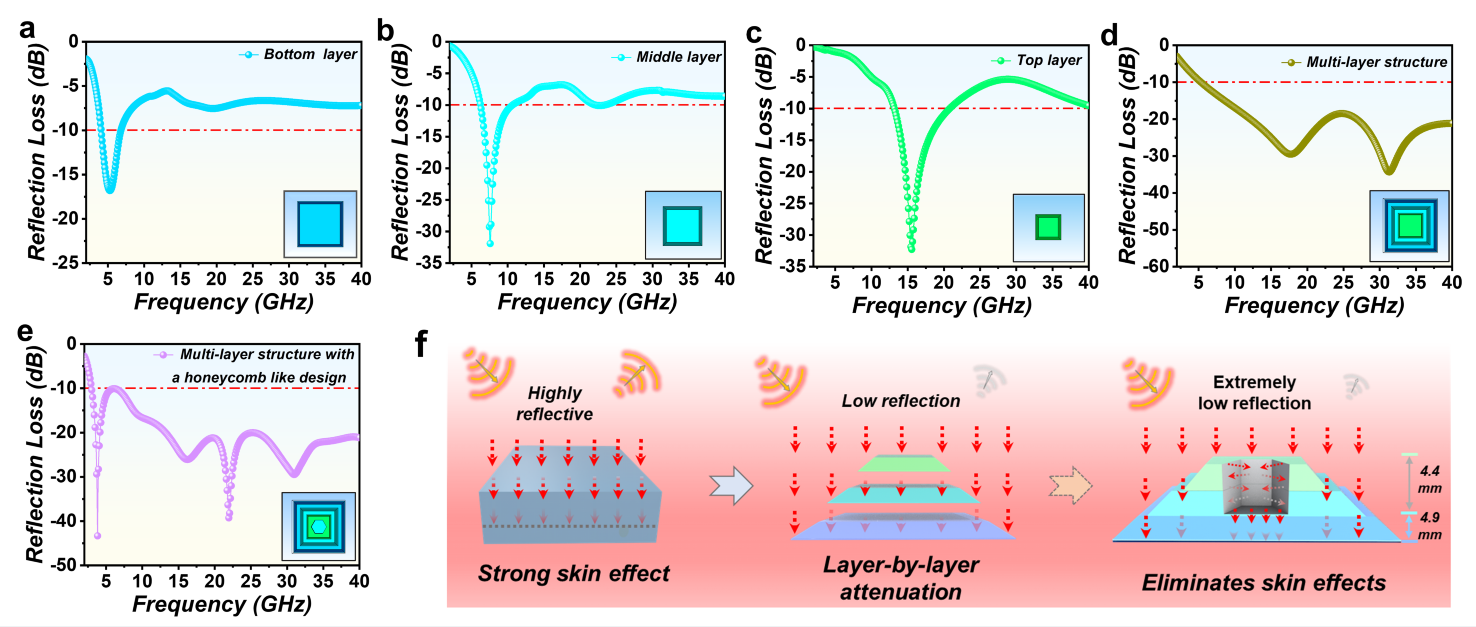
**

**Supplementary Fig. 13 Simulated EMW absorption performance of the proposed metamaterial structure.** **a** Bottom layer (S3), **b** Middle layer (S2), **c** Top layer (S1), **d** Multi-layer structure (top to bottom: S1-S2-S3) and **e** Multi-layer structure (top to bottom: S1-S2-S3) with a honeycomb perforation. **f** Basic rationale for the effectiveness of multilayer design.

It is evident that the single-layer metamaterials based on CuHT-FCIP-EP-S1~S3 exhibit certain electromagnetic responses within the low (2-6 GHz), middle (6-10 GHz), and high (10-20 GHz) frequency ranges, respectively. However, they are severely constrained by narrow bandwidth (**Supplementary Fig. 13a-c**). The combined structure of the three-layer slab significantly broadens the overall effective absorption bandwidth (EAB) of the metamaterial, achieving broadband absorption from 5.2 to 40 GHz. Additionally, two distinct resonance peaks appear on the absorption curve at 16.1 GHz and 32.2 GHz, mainly attributed to the thickness resonances at λ/2 and λ (with a thickness of 9.3 mm) of the metamaterial. (**Supplementary Fig. 13d**) Building upon this, a honeycomb perforation is incorporated on top of the metamaterial to expand the EAB further by introducing structural resonances, which effectively mitigates the skin effect ^24^ caused by the increase in dielectric constant and thickness of the material. As a result, another two distinct absorption peaks appear (3.82 GHz and 22.0 GHz) while the former two resonance peaks remain unchanged. (**Supplementary Fig. 13e-f**) This confirms the validity of our proposed novel metamaterial model.

**
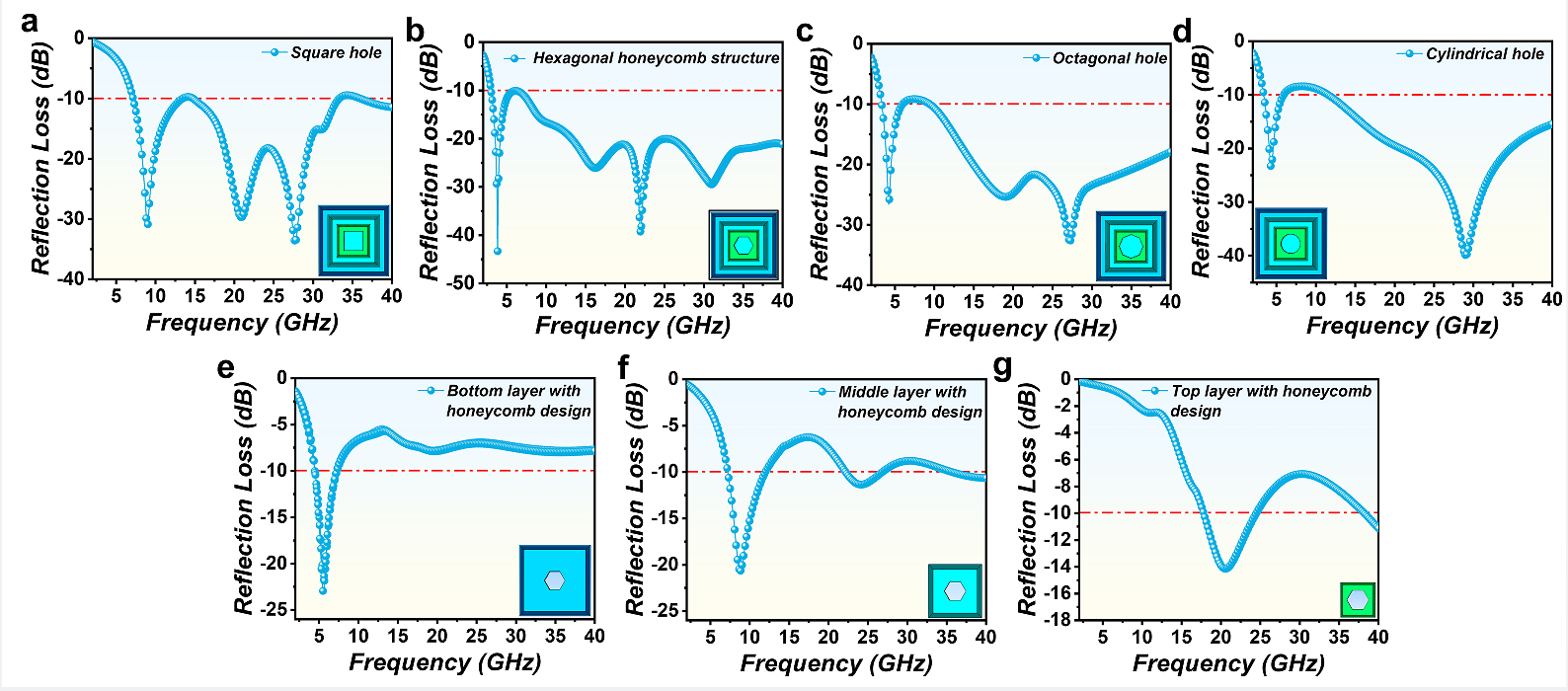
**

**Supplementary Fig. 14** **Simulation of reflection loss for metamaterials with different geometric perforation features.** **a** Square, **b** Hexagon, **c** Octagon and **d** Cylinder. **e-g** Single-layer hexagonal honeycomb perforation for bottom layer, middle layer and top layer with the same geometry parameters as the CuHT-FCIP-EP metamaterials.

The hexagonal honeycomb structure is a geometric structure with well-documented electromagnetic resonance effects. It possesses good symmetry (C3 symmetry) and spatial utilization efficiency. The primary purpose of introducing perforations is to facilitate the incidence of low-frequency (with longer wavelength) EMWs and inducing edge resonance of high-frequency EMWs. Therefore, selecting a geometric structure with the maximum cross-sectional area and retaining as many characteristic straight edges as possible is desired. To achieve this, we simulated the reflection curves of perforated structures with different numbers of side lengths (as shown in **Supplementary Fig. 14**). The results show that as the number of edges of the perforation structure increases, the characteristic absorption peak in the low-frequency region gradually approaches 2 GHz. This indicates that increasing the cross-sectional area facilitates the entrance of low-frequency EMWs and further causes losses. However, as the number of edges increases, the length of the edge decreases (for a circle, the side length can be considered infinitely small), thereby weakening the edge resonance effect of high-frequency EMWs. Therefore, ultimately, we choose the hexagonal perforation structure.

Besides, honeycomb structures are widely acknowledged in engineering for their exceptional mechanical load-bearing capabilities. This is attributed to their inherent ability to offer higher stiffness and strength with minimal weight. The design of honeycomb structures, inspired by nature, enables them to distribute loads uniformly in all directions while utilizing minimal material. As a result, honeycomb structures find extensive applications in aerospace, vehicle manufacturing, and various other fields that demand lightweight yet robust solutions. In this work, with the help of honeycomb perforation, the bulk density of the metamaterial is only 0.89 g/cm^3^ leading to an excellent specific compressive strength (201.01 MPa·m^3^·kg^-1^).


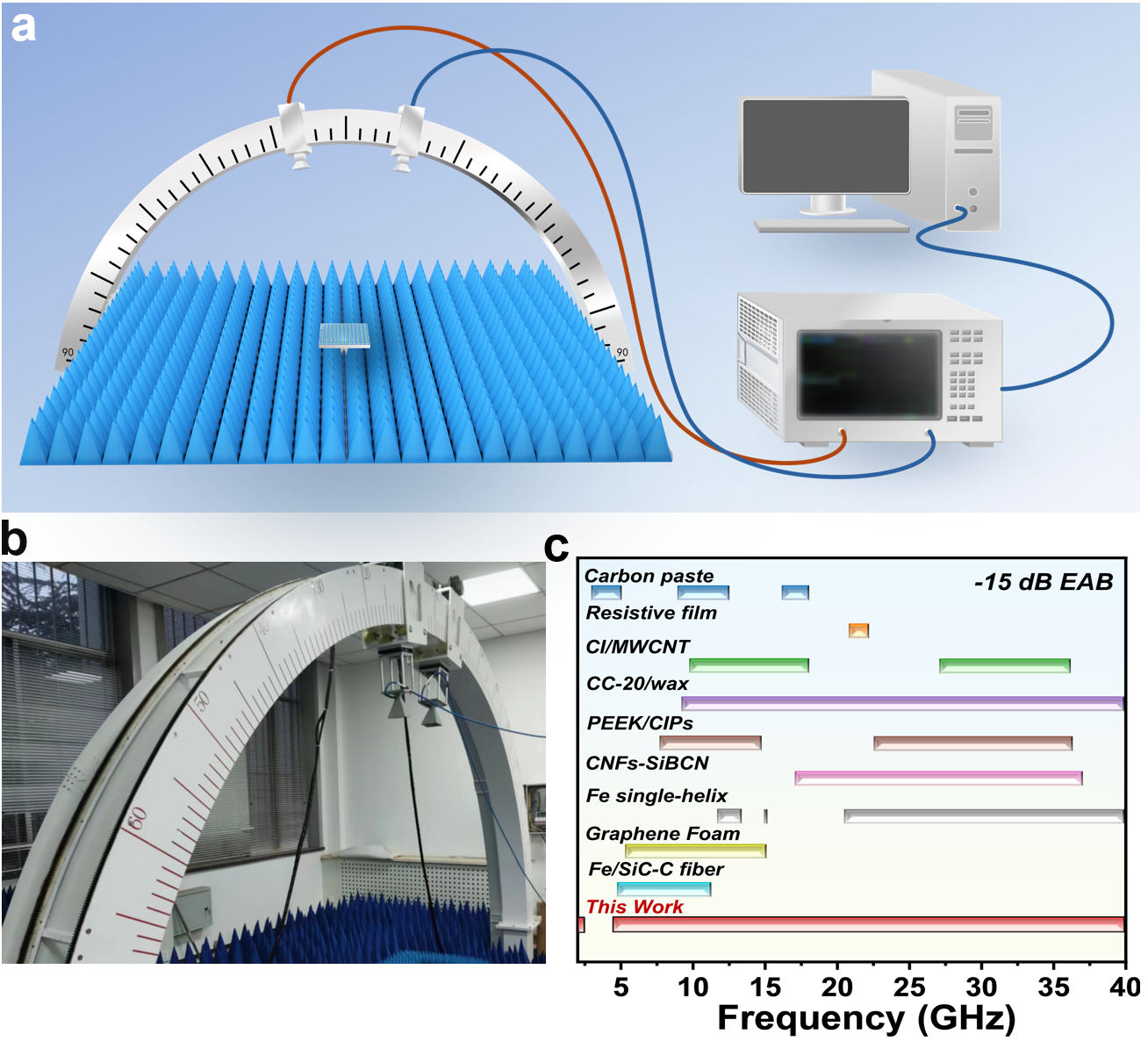


**Supplementary Fig. 15** **Far-field absorption verification of metamaterial absorbers. a** Schematic of the far field test setup, **b** Device photo. **c** Comparison of far field performance between CuHT-FCIP based metamaterial absorber and some typical 3D metamaterials (EAB, RL<-15 dB), and the relevant data ^25-33^ are summarized in **Supplementary Table 4**.

**Supplementary Table 5** Comparison of absorption performance with recently reported 3D metamaterials.

| **Sample** | **Compone-nt** | **EAB (GHz)**  **<-10 dB** | **EAB (GHz)**  **<-15 dB** | **EAB (<-10 dB)**  **(S-C band)** | **Thick-ness (mm)** | **RL_min_ (dB)** | **Compre-ss Strength (MPa)** | **Den-sity Test** | **Ref.** |
| --- | --- | --- | --- | --- | --- | --- | --- | --- | --- |
| Carbon coated corrugated structure | Carbon paste | 2.31-18.00 | 3.00-5.00, 9.00-12.50, 16.20-18.00 | 5.69 | 18.00 | -25.93 | No | No | 25 |
| 3D resistance film structure | Resistive film | 3.90-26.20 | 20.80-22.00 | 4.10 | 11.00 | -15.90 | No | No | 26 |
| Spherical CI/MWCNT epoxy resin | CI/MWCNT | 2.00-2.36, 6.54-19.36, 19.89-21.57, 25.10-40.00 | 9.78-18.00, 27.14-36.11 | 1.82 | 7.00 | -54.00 | No | No | 27 |
| 3D printed shell with CC-20/wax patterns | CC-20/wax | 7.81-40.00 | 9.02-40.00 | 0.19 | 11.00 | -34.50 | No | No | 28 |
| PEEK/CIPs metastructure | PEEK/CIPs | 5.10-16.80,18.25-40.00 | 7.70-14.70, 22.60-36.30. | 2.90 | 10.00 | -30.50 | 8.46 | No | 29 |
| CNFs-SiBCN-based metamaterial | CNFs-SiBCN | 7.60-40.00 | 17.00-37.00 | 0.40 | 5.00 | -46.00 | No | No | 30 |
| Nested triple-helix | Fe single-helix | 11.50-13.75,  15.00-15.50,  18.70-40.00 | 11.67-13.30  15.00-15.05  20.50-40.00 | 0.00 | 15.00 | -42.50 | No | No | 31 |
| Graphene foam | Graphene Foam | 4.10-18.00, 26.50-29.80 | 5.40-15.00 | 0.90 | 10.00 | -35.00 | No | No | 32 |
| Fe/SiC-C fiber superstructure | Fe/SiC-C fiber | 2.80-18.00 | 4.80-11.20 | 5.20 | 8.45 | -24.00 | No | No | 33 |
| **CuHT-FCIP-EP metamaterial** | **CuHT-FCIP 2D/2D assembly** | **2.00-40.00** | **2.00-2.35**  **4.50-40.00** | **6.00** | **9.30** | **-51.00** | **201.01** | **0.89** | **This work** |


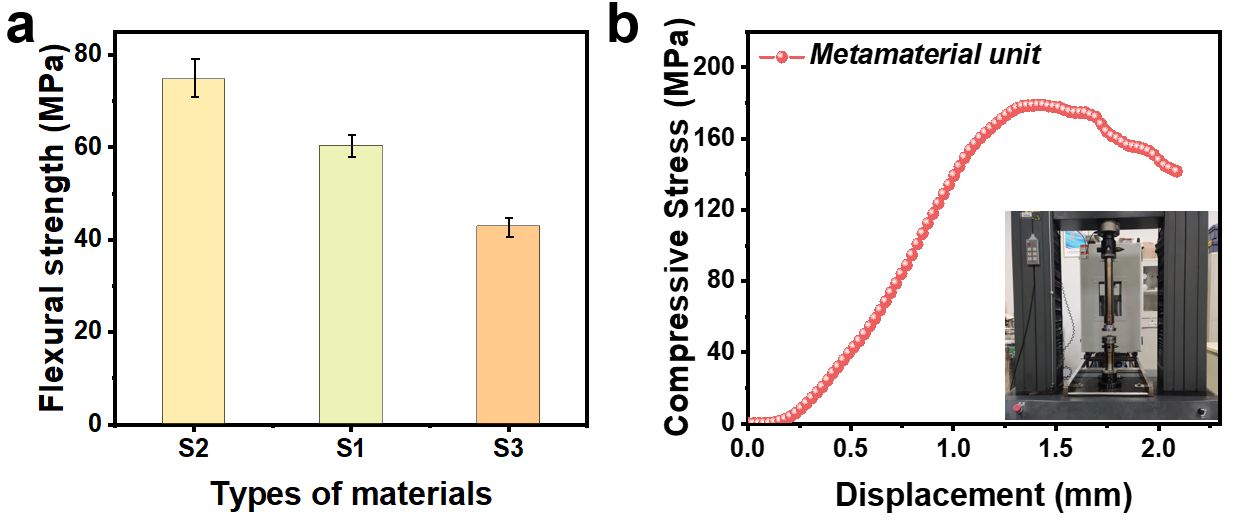


**Supplementary Fig. 16** Mechanical properties of CuHT-FCIP-EP composites. **a** Stress–strain curves of S1, S2, S3 under three-point bending test. **b** Stress–displacement curve of the metamaterial absorber (2×2 unit structures).

**Supplementary References**

1. Guayaquil-Sosa, F. et al. Photocatalytic hydrogen production using mesoporous TiO_2_ doped with Pt. *Appl Catal B Environ* **211**, 337-348 (2017).

2. Kresse, G. et al. Efficient iterative schemes for ab initio total-energy calculations using a plane-wave basis set. *Phys Rev B* **54**, 11169-11186 (1996).

3. Perdew, J. P. et al. Generalized gradient approximation made simple. *Phys Rev Lett* **77**, 3865-3868 (1996).

4. Kresse, G. et al. From ultrasoft pseudopotentials to the projector augmented-wave method. *Phys Rev B* **59**, 1758-1775 (1999).

5. Blochl, P. E. Projector augmented-wave method. *Phys Rev B* **50**, 17953-17979 (1994).

6. Monkhorst, H. J. et al. Special points for brilllouin-zone integrations. *Phys Rev B* **13**, 5188-5192 (1976).

7. Grimme, S. et al. A consistent and accurate ab initio parametrization of density functional dispersion correction (DFT-D) for the 94 elements H-Pu. *J Chem Phys* **132**, 154104 (2010).

8. Grimme, S. et al. Effect of the Damping Function in Dispersion Corrected Density Functional Theory. *J Comput Chem* **32**, 1456-1465 (2011).

9. Wang, V. et al. VASPKIT: A user-friendly interface facilitating high-throughput computing and analysis using VASP code. *Comput Phys Commun* **267**, 108033 (2021).

10. Low, K. H. et al. Highly conducting two-dimensional copper(I) 4-hydroxythiophenolate network. *Chem Commun* **46**, 7328-7330 (2010).

11. Cheng, Y. et al. Rationally regulating complex dielectric parameters of mesoporous carbon hollow spheres to carry out efficient microwave absorption. *Carbon* **127**, 643-652 (2018).

12. Miao, P. et al. A Two-Dimensional Semiconductive Metal-Organic Framework for Highly Efficient Microwave Absorption. *Chin J Chem* **40**, 467-474 (2022).

13. Xie, Y. et al. Efficient electromagnetic wave absorption performances dominated by exchanged resonance of lightweight PC/Fe_3_O_4_@PDA hybrid nanocomposite. *Chem Eng J* **457**, 141205 (2023).

14. Yang, K. K. et al. Transforming in-situ grown chitosan/ZIF-67 aerogels into 3D N-doped Co/CoO/carbon composites for improved electromagnetic wave absorption. *J Alloys Compd* **936**, 168195 (2023).

15. Jia, H. et al. 1D CNTs assembled MOF-derived hollow CoSe_2_@N-doped carbon constructed high-efficiency electromagnetic wave absorbers. *Carbon* **215**, 118400 (2023).

16. Ban, Q. et al. Polymerization-induced assembly-etching engineering to hollow Co@N-doped carbon microcages for superior electromagnetic wave absorption. *Carbon* **215**, 118506 (2023).

17. Zhu, H. et al. Cu/NC@Co/NC composites derived from core-shell Cu-MOF@Co-MOF and their electromagnetic wave absorption properties. *J Colloid Interface Sci* **613**, 182-193 (2022).

18. Kong, M. et al. Porous magnetic carbon CoFe alloys@ZnO@C composites based on Zn/Co-based bimetallic MOF with efficient electromagnetic wave absorption. *J Colloid Interface Sci* **604**, 39-51 (2021).

19. Jia, Z. et al. Tunable Co/ZnO/C@MWCNTs based on carbon nanotube-coated MOF with excellent microwave absorption properties. *J Mater Sci Technol* **127**, 153-163 (2022).

20. Wang, Y. et al. MOF-derived nanoporous carbon/Co/Co_3_O_4_/CNTs/RGO composite with hierarchical structure as a high-efficiency electromagnetic wave absorber. *J Alloys Compd* **846**, 156215 (2020).

21. Wang, Y. et al. Pearl necklace-like CoMn-based nanostructures derived from metal-organic frames for enhanced electromagnetic wave absorption. *Carbon* **188**, 254-264 (2022).

22. Wang, Y. et al. Magnetic CoFe alloy@C nanocomposites derived from ZnCo-MOF for electromagnetic wave absorption. *Chem Eng J* **383**, 123096 (2020).

23. Yudistira, H. T. et al. The Study of the Altering Substrate Refractive Index on Single Symmetry Split-Ring Resonator Unit Cell Metamaterial. *Plasmonics* **16**, 1849-1853 (2021).

24. Gong, C. C. et al. Utilizing Se vacancies as electronic traps to synergize impedance matching and dipole polarization with ultrathin strategy to boost Fe-Se electromagnetic wave absorption. *Chem Eng J* **480**, 147793 (2024).

25. Huang, H. et al. Broadband radar absorbing performance of corrugated structure. *Compos Struct* **253**, 112809 (2020).

26. Shen, Y. et al. An extremely wideband and lightweight metamaterial absorber. *J Appl Phys* **117**, 224503 (2015).

27. Huang, Y. X. et al. Multi-scale design of electromagnetic composite metamaterials for broadband microwave absorption. *Compos Sci Technol* **162**, 206-214 (2018).

28. Song, W. L. et al. Constructing Repairable Meta-Structures of Ultra-Broad-Band Electromagnetic Absorption from Three-Dimensional Printed Patterned Shells. *ACS Appl Mater Interfaces* **9**, 43179-43187 (2017).

29. Duan, Y. B. et al. A wide-angle broadband electromagnetic absorbing metastructure using 3D printing technology. *Mater Des* **208**, 109900 (2021).

30. Liu, H. Q. et al. Additive manufacturing of nanocellulose/polyborosilazane derived CNFs-SiBCN ceramic metamaterials for ultra-broadband electromagnetic absorption. *Chem Eng J* **433**, 133743 (2022).

31. He, Z. et al. Ultrawide bandwidth and large-angle electromagnetic wave absorption based on triple-nested helix metamaterial absorbers. *J Appl Phys* **127**, 174901 (2020).

32. Zhang, Y. et al. Broadband and Tunable High-Performance Microwave Absorption of an Ultralight and Highly Compressible Graphene Foam. *Adv Mater* **27**, 2049-2053 (2015).

33. Zhao, Y. J. et al. Development of multiscale Fe/SiC-C fibrous composites for broadband electromagnetic and acoustic waves absorption. *Compos Part B Eng* **250**, 110454 (2023).
